# Fate of the Discoverers of Anæsthesia

**Published:** 1880-12

**Authors:** 


### FATE OF THE DISCOVERERS OF ANÆSTHESIA.

It is worth while remarking—leaving the partisans of Wells, Morton, Long, and Jackson to settle for themselves to whom should be ascribed the discovery of artificially induced insensibility which has saved so much pain and has so widely enlarged the boundries of surgical activity—that the fate of nearly all the American claimants was tragic. Long was the happiest. He died, comparatively little known, in 1878, a poor man, though now his statue, with that of Oglethorpe, will represent Georgia in the National Gallery at the Capitol. Morton, having been reduced to poverty during the long twelve years in which he endeavored to wring from Congress and the Courts recognition of his rights, died suddenly in New York city in 1868, of cerebral congestion brought on, it is said, by reading a work attacking his claims. Wells' mind failed in the fierce controversy, and after his arrest in New York in 1848 for throwing vitrol on women's clothing in the streets he destroyed himself, while Jackson's mind had for some years been clouded between agitation and dissappointment. It is not without truth that Dr Hayward said of the discovery that "the only spot in Christendom in which it was received with coldness was in our own country." How much more fortunate was Simpson, whose introduction of chloroform won for him a baronetcy, the highest honors in his profession, a statue in Edinburg and a memorial bust in Westminster Abbey! —*Med. Library Jour.*

